# Bipolar/rod-shaped microglia are proliferating microglia with distinct M1/M2 phenotypes

**DOI:** 10.1038/srep07279

**Published:** 2014-12-02

**Authors:** Wing Yip Tam, Chi Him Eddie Ma

**Affiliations:** 1 Department of Biomedical Sciences, City University of Hong Kong, Tat Chee Avenue, Hong Kong; 2 Centre for Biosystems, Neuroscience, and Nanotechnology, City University of Hong Kong, Tat Chee Avenue, Hong Kong; 3 State Key Laboratory in Marine Pollution, City University of Hong Kong, Tat Chee Avenue, Hong Kong

## Abstract

Microglia are generally considered the resident immune cells in the central nervous system (CNS) that regulate the primary events of neuroinflammatory responses. Microglia also play key roles in repair and neurodegeneration of the CNS after injury. Recent studies showed that trains of bipolar/rod-shaped microglia align end-to-end along the CNS injury site during the initial recovery phase. However, the cellular characteristics of bipolar/rod-shaped microglia remain largely unknown. Here, we established a highly reproducible *in vitro* culture model system to enrich and characterize bipolar/rod-shaped microglia by simply generating multiple scratches on a poly-d-lysine/laminin-coated culture dish. Trains of bipolar/rod-shaped microglia formed and aligned along the scratches in a manner that morphologically resembled microglial trains observed in injured brain. These bipolar/rod-shaped microglia were highly proliferative and expressed various M1/M2 markers. Further analysis revealed that these bipolar/rod-shaped microglia quickly transformed into amoeboid microglia within 30 minutes of lipopolysaccharide treatment, leading to the upregulation of pro-inflammatory cytokine gene expression and the activation of Jak/Stat. In summary, our culture system provides a model to further characterize this highly dynamic cell type. We suggest that bipolar/rod-shaped microglia are crucial for repairing the damaged CNS and that the molecular mechanisms underlying their morphological changes may serve as therapeutic biomarkers.

Microglia are mononuclear phagocytes located in the CNS, and their functions include antigen presentation, cytokine and chemokine production, neurogenesis-promoting neurotrophic factor secretion^1^,^2^ and phagocytosis to remove dead cells and pathogens as a component of innate immune responses^3^. Microglial activation is associated with many neurological conditions, such as inflammatory brain diseases and brain injuries^4^,^5^. The activation states of microglia can be loosely categorized into “classical activation” (M1) and “alternative activation” (M2) as commonly described for macrophages^6^. The microglial M1/M2 classification paradigm, which was originally adopted for macrophages, remains controversial in part due to the partially overlapping expression of macrophage M1 and M2 markers in microglia under physiological or pathological conditions^7^,^8^,^9^,^10^ (see Discussion for more details). In general, an M1 phenotype represents pro-inflammatory activity, including the production of pro-inflammatory cytokines, such as tumor necrosis factor (Tnf) and interleukin-1b (Il-1b). M2 microglia exhibit an anti-inflammatory phenotype by expressing Il-10 and transforming growth factor-β (Tgf-β), which facilitate wound healing^8^,^11^,^12^.

Microglia are morphologically dynamic cells whose morphological changes are closely associated with their functional activities^13^,^14^,^15^. Most previous studies focused on ramified and amoeboid microglia largely based on the finding that they are the most common forms of microglia found in the brain at different developmental stages^5^,^15^. Ramified microglia act as surveying cells by actively sensing the surrounding environment via dynamic processes^3^,^13^,^16^,^17^. Amoeboid microglia are highly motile and participate in phagocytosis^14^,^15^. The availability of a well-defined and reproducible culture system to examine ramified and amoeboid microglia would greatly enhance the understanding of these forms of microglia. The ramified or amoeboid morphology can be maintained by culturing microglia on fibronectin-coated and laminin-coated surfaces, respectively^18^. However, bipolar/rod-shaped microglia remain very poorly understood and characterized, primarily due to the lack of a well-defined culture model system and the difficulties in imaging these highly dynamic live microglia in deep brain tissues^19^,^20^. Recent studies have shown that bipolar/rod-shaped microglia transiently form trains of cells aligned end-to-end at the damaged site after brain injury^19^,^20^,^21^. Bipolar/rod-shaped microglia are also found in the cerebral cortex of patients with neural disorders such as Alzheimer's disease, subacute sclerosing panencephalitis, lead encephalopathy and viral encephalitis^22^,^23^,^24^. Despite progress in characterizing the functional role of bipolar/rod-shaped microglia, their fundamental molecular and cellular properties remain largely unknown.

In this study, we established a simple and highly reproducible cell culture system to examine bipolar/rod-shaped microglia. We found that trains of bipolar/rod-shaped microglia aligned end-to-end along the scratched area of a poly-d-lysine (PDL)- and laminin-coated surface in close resemblance to the microglial alignment observed after brain injury *in vivo*^19^,^20^,^21^. Our time-lapse live cell imaging study showed that the scratched areas attracted colonies of microglia. These highly proliferative (proliferating cell nuclear antigen [PCNA]-positive) bipolar/rod-shaped microglia displayed differential expression of M1 and M2 markers, suggesting that these microglia exhibited distinct M1/M2 phenotypes. Following treatment with the classic M1 stimulus lipopolysaccharide (LPS), bipolar/rod-shaped microglia were readily transformed into the amoeboid form, including the upregulation of *Tnf* and *Il-1b* expression in approximately 30 minutes. Therefore, we propose that our culture system serves as a valuable tool for the further characterization and functional analysis of bipolar/rod-shaped microglia.

## Results

### Bipolar/rod-shaped microglia align in parallel with the direction of the scratch

**Figure 1:**
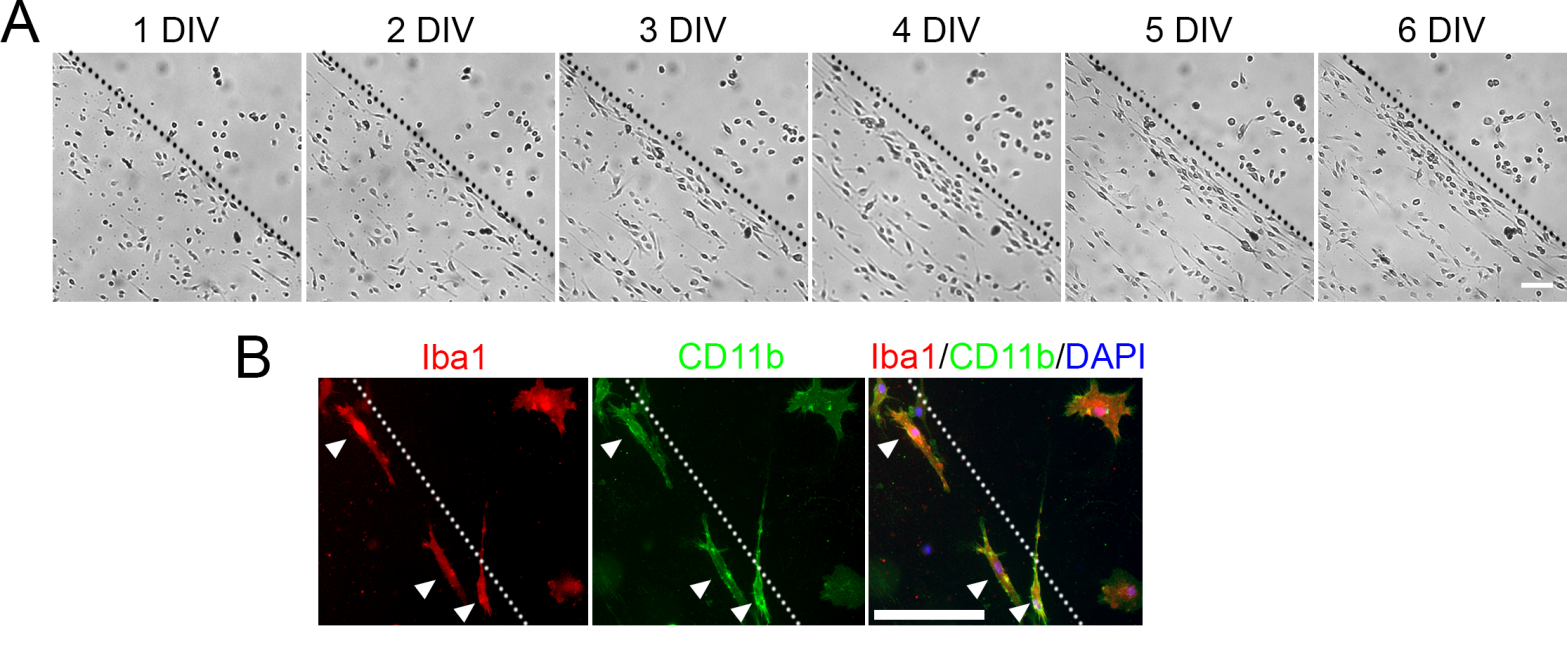
Bipolar/rod-shaped microglia aligned along the scratch of the coated surface *in vitro*. (a) Bipolar/rod-shaped microglia colonized in the scratched area of a PDL/laminin-coated surface. The cell processes were randomly aligned at 1 DIV and then displayed a more synchronized alignment along the scratch at 6 DIV. (b) Both bipolar/rod-shaped (arrows) and amoeboid microglia expressed the microglial markers Iba1 (red) and CD11b (green). The dotted line indicates the boundary between the scratched (left to the line) and non-scratched areas. Scale bars: A: 100 μm; B: 50 μm.

Mouse primary microglia were seeded on PDL/laminin-coated culture chambers with or without scratches on the surface. Interestingly, we found that most of the microglia in the scratched area extended bipolar processes in random directions beginning at 1 day *in vitro* (DIV) (Fig. 1a). Then, the microglia became densely packed, and their processes aligned in parallel with the direction of the scratch until a stable alignment was attained at 6 DIV. In the non-scratched area, most, if not all, of the microglia adopted an amoeboid morphology (Fig. 1a), as described previously^18^. To confirm that the bipolar/rod-shaped microglia maintained their microglial identity on the scratched surface, we performed immunostaining for the classical microglial markers Iba1^25^ and CD11b^26^,^27^. The bipolar/rod-shaped and amoeboid microglia expressed similar levels of Iba1 and CD11b (Fig. 1b). Our data indicated that scratching the PDL/laminin-coated surface promoted the formation of bipolar/rod-shaped microglia without affecting their specificity, whereas the non-scratched surface supported the growth of amoeboid microglia.

### Bipolar/rod-shaped microglia are highly proliferative

**Figure 2:**
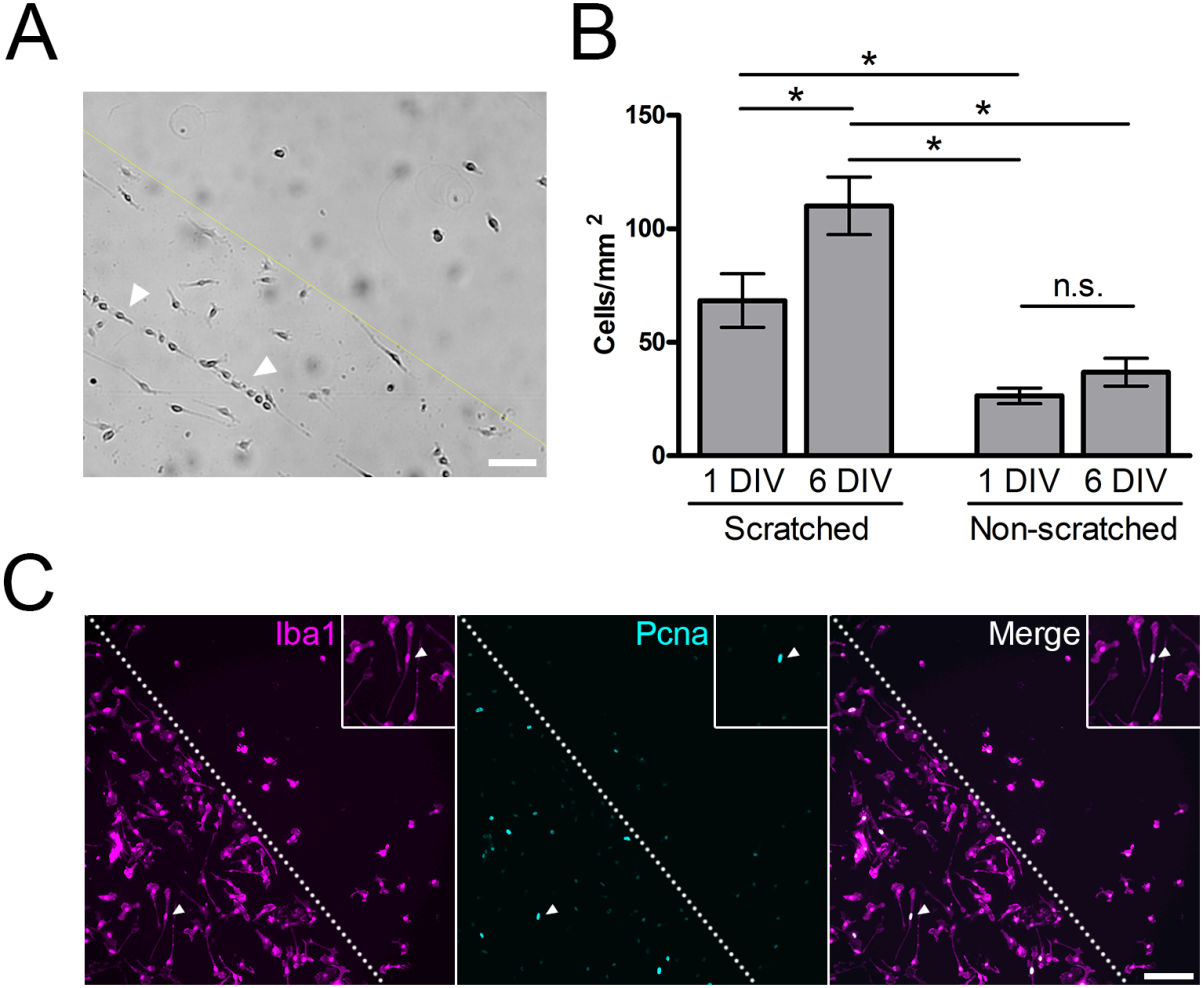
Bipolar/rod-shaped microglia were highly proliferative. (a) Trains of microglia formed along the scratch (left of the dotted line). (b) The density of the microglia significantly increased in the scratched area from 1 DIV to 6 DIV (*P < 0.05 by ANOVA) but not in the non-scratched area (n = 3). (c) Strong signal of the proliferation marker PCNA was localized to the nucleus of the microglia primarily in the scratched area (left of the dotted line). Most of the PCNA^+^ cells were bipolar/rod-shaped (inset). Scale bar: 100 μm.

Recent studies showed that trains of bipolar/rod-shaped microglia formed within 24 hours and peaked at 7 days at the site of injury after brain damage^19^,^20^. Here, in our *in vitro* system, we also observed trains of microglial colonies along the scratches within 24 hours after cell seeding (Fig. 2a). The aligned bipolar/rod-shaped microglia became densely packed in the scratched area compared to the non-scratched area at 6 DIV (Fig. 1a). In the scratched area, the cell density nearly doubled from 68.4 ± 11.79 cells/mm^2^ at 1 DIV to 110.1 ± 12.70 cells/mm^2^ at 6 DIV (p<0.05) (Fig. 2b). In contrast to the scratched area, there was no significant difference in cell density between 1 DIV (26.4 ± 3.40 cells/mm^2^) and 6 DIV (36.9 ± 6.12 cells/mm^2^) in the non-scratched area (Fig. 2b). This finding suggested that bipolar/rod-shaped microglia exhibit a higher proliferative capacity than amoeboid microglia. Therefore, we examined the expression of the proliferating cell marker PCNA in the microglial cell cultures. The majority of prominent PCNA-positive immunoreactivity was localized to the nuclei of bipolar/rod-shaped microglia in the scratched area (Fig. 2c), suggesting that these bipolar/rod-shaped microglia were highly proliferative.

### The scratched area promotes microglial colonization

**Figure 3:**
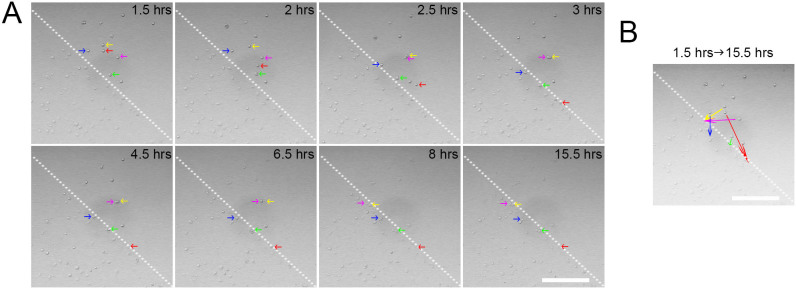
Scratched area attracted colonies of microglia. (a) Time-lapse live cell imaging showed the migration of microglia (depicted by arrows with different colors) from the non-scratched area to the scratched area. (b) Simplified migratory paths of the microglia from 1.5 hours to 15.5 hours after cell seeding. Scale bars: 250 μm.

A higher cell density was observed at 1 DIV in the scratched area (68.4 ± 11.79 cells/mm^2^) than in the non-scratched area (26.4 ± 3.40 cells/mm^2^). It is very likely that the microglia migrated toward scratches as opposed to the proliferation of colonized microglia in the scratched area, as it took five days for the number of microglia to double (Fig. 2b). To test this hypothesis, we performed time-lapse imaging of live microglia, which illustrated that the microglia migrated toward scratches during the initial 14 hours (Fig. 3 and Supplementary Video S1 and S2). The observed clustering of microglia in the scratched area beginning at 1 DIV occurred due to the active migration of microglia from the non-scratched area (Fig. 3) rather than the proliferation of colonized microglia in the scratched area. These results demonstrated that the scratches damaged the extracellular matrix (laminin) on the coating surface, which attracted the colonization of microglia, further suggesting that our *in vitro* system mimicked the *in vivo* brain injury conditions.

### Differential expression of M1 and M2 markers in bipolar/rod-enriched microglia

**Figure 4:**
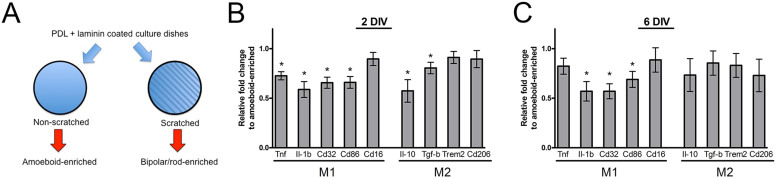
Reduced expression of M1 and M2 markers in bipolar/rod-shaped microglia. (a) Schematic diagram illustrating the amoeboid-enriched and bipolar/rod-shaped enriched cultures. (b) Reduced expression of selected M1 and M2 markers in the bipolar/rod-enriched microglia cultures compared with the amoeboid-enriched cultures at 2 DIV (n = 5 to 6). (c) This reduced M1 marker expression was generally maintained, whereas M2 marker expression increased at 6 DIV (n = 5 to 6). *P < 0.05 based on Student's *t*-test.

To determine the activation state of the bipolar/rod-shaped microglia, we performed qPCR to assess the mRNA levels of genes associated with M1 or M2 activation. We compared the gene expression of microglia in non-scratched (amoeboid-enriched) and scratched (bipolar/rod-enriched) PDL/laminin-coated culture dishes (Fig. 4a). Amoeboid microglia are known to produce pro-inflammatory cytokines and, thus, are assumed to be M1-activated^8^,^28^,^29^,^30^,^31^. At 2 DIV, we found that four out of the five analyzed M1 markers were significantly downregulated, including *Tnf* (0.73-fold), *Il-1b* (0.59-fold), *Cd32* (0.66-fold) and *Cd86* (0.66-fold), in bipolar/rod-enriched cultures compared with amoeboid microglia (Fig. 4b). Interestingly, two out of the four examined M2 markers, *Il-10* (0.57-fold) and *Tgf-β* (0.80-fold), were also significantly down-regulated (Fig. 4b). At 6 DIV, three out of the four M1 examined markers remained down-regulated, with the exception of *Tnf* (Fig. 4c). In contrast, the expression of all M2 markers was restored to the levels in amoeboid microglia (Fig. 4c). Taken together, these data indicated that bipolar/rod-shaped microglia exhibit reduced expression of M1 and M2 markers during early stages of train formation, and M2 marker expression increased after stable microglia alignment occurred.

### M1 activation of bipolar/rod-shaped microglia by LPS

Based on our qPCR analysis, we found that bipolar/rod-shaped microglia exhibited reduced expression of M1 markers, suggesting that these cells may be quiescent with respect to pro-inflammatory cytokine production. Therefore, we examined whether 1) bipolar/rod-shaped microglia are susceptible to M1 activation by LPS, which is a widely used M1 stimulus that is known to trigger pro-inflammatory cytokine production^11^,^32^,^33^,^34^,^35^,^36^,^37^, and 2) bipolar/rod-shaped microglia must change morphologically before they can be activated, as bipolar/rod-shaped microglia have been suggested to represent a transitional stage between the activated and deactivated states^38^.

**Figure 5:**
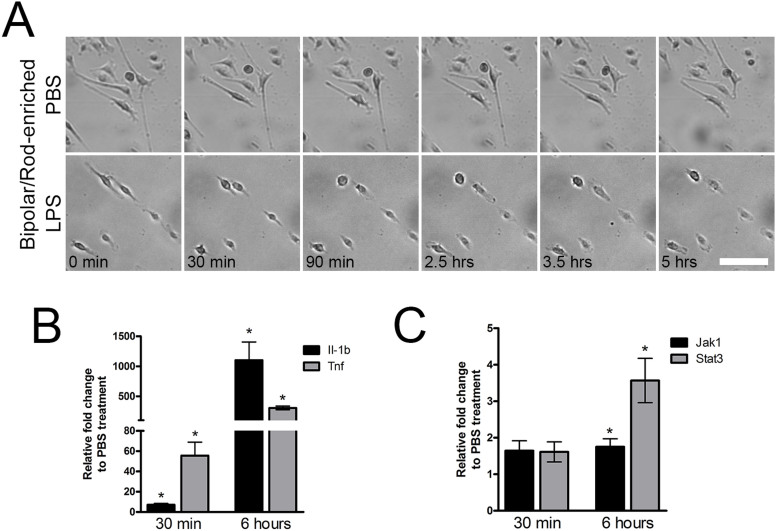
Bipolar/rod-shaped microglia rapidly responded to M1 activation using LPS. (a) Bipolar/rod-shaped microglia converted to an amoeboid morphology in response to LPS stimulation, but not PBS treatment, in 30 minutes. (b) qPCR showed significant up-regulation of *Il-1b* and *Tnf* after 30 minutes and 6 hours of LPS stimulation compared with PBS treatment (n = 4). (c) The qPCR results showed up-regulation of *Jak1* and *Stat3* shortly after 30 minutes that increased significantly after 6 hours of LPS treatment compared with PBS treatment (n = 4). *P < 0.05 based on Student's *t*-test.

The bipolar/rod-shaped microglia converted into the amoeboid form within 30 minutes in the presence of LPS, and their amoeboid morphology was maintained for at least 5 hours (Fig. 5a). In contrast, no morphological change was observed in the microglial cultures treated with the vehicle control PBS (Fig. 5a). Next, we performed qPCR to analyze the expression of the pro-inflammatory cytokines *Tnf* and *Il-1b* at 30 minutes and 6 hours post-LPS treatment. Remarkably, *Il-1b* expression was increased by 7-fold at 30 minutes and by greater than 1,100-fold at 6 hours compared with the corresponding controls; alternatively, *Tnf* was up-regulated by 56-fold and greater than 300-fold, respectively (Fig. 5b). We also examined the expression of *Jak1* and *Stat3* because the Jak1/Stat3 pathway is a well-known modulator of pro-inflammatory cytokine expression in microglia^39^,^40^,^41^,^42^. The expression of both *Jak1* and *Stat3* was up-regulated by approximately 1.6-fold at 30 minutes. After 6 hours, Jak1 and Stat3 expression was significantly increased by 1.8-fold and 3.6-fold, respectively (Fig. 5c). Moreover, we examined the response of amoeboid microglia to LPS as a positive control. The amoeboid microglia maintained their morphology after LPS stimulation or PBS treatment (Supplementary Figure S1a). Consistent with other studies, qPCR analysis indicated that both *Tnf* and *Il-1b* were significantly up-regulated after 30 minutes and 6 hours of LPS stimulation compared with PBS treatment (Supplementary Figure S1b). Taken together, our results showed that bipolar/rod-shaped microglia rapidly responded to LPS stimulation by transforming into the amoeboid form and by inducing the gene expression of key pro-inflammatory cytokines via the up-regulation of *Jak1* and *Stat3*.

## Discussion

In the current study, we developed a simple, cost-effective, and highly reproducible culture method for the enrichment of bipolar/rod-shaped microglia by simply generating multiple scratches on a PDL/laminin-coated surface, resulting in the formation of trains of bipolar/rod-shaped microglia exclusively in the scratched area. Using this culture method, we have characterized these bipolar/rod-shaped microglia and revealed that they (1) are highly proliferative, (2) express reduced mRNA levels of pro-inflammatory M1 and anti-inflammatory M2 markers compared with amoeboid microglia, and (3) transform into the amoeboid form, including increased production of pro-inflammatory cytokines *Tnf* and *Il-1b*, via the up-regulation of *Jak1* and *Stat3* expression.

Bipolar/rod-shaped microglia were first documented nearly a century ago^19^,^43^. However, little information is available regarding this microglia type. Several studies provided further insights into bipolar/rod-shaped microglia, especially their association with neuropathologies and brain injuries^19^,^20^,^22^,^24^. Transient accumulation of bipolar/rod-shaped microglia trains has been observed at the site of injury during the early phase of brain damage^19^,^20^,^21^, which may be crucial for minimizing further damage and facilitating repair, as reducing the number of proliferating microglia results in more severe damage to the cortex after ischemic insult^44^,^45^. More importantly, the bipolar/rod-shaped microglia trains primarily colocalized with neurons and axons but not with other glia after brain injury^19^,^20^. Our study is consistent with other *in vivo* studies showing prominent proliferation of bipolar/rod-shaped microglia^14^,^24^,^46^, suggesting that the formation of highly proliferative bipolar/rod-shaped microglia trains may represent a critical step for increasing the number of microglia at the site of injury. Subsequently, bipolar/rod-shaped microglia rapidly transform into an amoeboid morphology and exhibit phagocytic activity, further enhancing the phagocytosis of dead or dying neurons to minimize brain damage.

Persistent production of pro-inflammatory cytokines by microglia results in chronic neuroinflammation and is associated with the development of neurodegenerative diseases such as Parkinson's disease and Alzheimer's disease^47^,^48^,^49^. Our data showed that bipolar/rod-shaped microglia expressed lower levels of the pro-inflammatory cytokines *Tnf* and *Il-1b*, implying that bipolar/rod-shaped microglia do not contribute to inflammation-induced neurodegeneration. However, the expression of the anti-inflammatory cytokines *Il-10* and *Tgf-β* in bipolar/rod-shaped microglia increased from 2 DIV to 6 DIV. Il-10 is known to inhibit pro-inflammatory cytokine production by microglia^50^. Taken together, bipolar/rod-shaped microglia might exert neuroprotective effects by producing greater amounts of anti-inflammatory cytokines than pro-inflammatory cytokines at the site of brain injury. The balance between pro-inflammatory and anti-inflammatory cytokine expression determines the extent of recovery from brain injury and neurodegenerative disease progression^51^,^52^.

Microglia and macrophages exhibit distinct expression profiles of M1 and M2 markers, possibly due to their different origins^12^. Accumulating evidence suggests that the concept of microglial classification into M1 and M2, which was originally adopted for macrophages, is becoming increasingly controversial^6^,^7^,^12^. Studies have shown that some M1 or M2 markers are expressed only in macrophages and not in microglia. For instance, human M2-activated microglia did not express M2 macrophage markers, such as Arg1, Chi3l3, CD23, CD163 and CD206^7^,^9^. During postnatal brain development, microglia express both M1 and M2 markers, suggesting that immature microglia do not commit to either the M1 or M2 phenotype^8^. CCL2, a chemoattractant protein that triggers CNS inflammation, induces the expression of both pro-inflammatory and anti-inflammatory responses in microglia, thereby exhibiting neither an M1- nor an M2-skewed phenotype^10^. This finding agrees well with our data showing that naive bipolar/rod-shaped microglia did not exclusively express M1 or M2 markers but could be readily transformed into an M1 phenotype shortly after activation using LPS. Surprisingly, the detection of both LPS-induced morphological changes and the up-regulation of pro-inflammatory cytokine/*Jak1*/*Stat3* gene expression occurred within approximately 30 minutes. Therefore, we suggest that bipolar/rod-shaped microglia do not participate in pro-inflammatory cytokine production but can rapidly transform into an active form and produce high levels of pro-inflammatory cytokines upon LPS stimulation.

**Figure 6:**
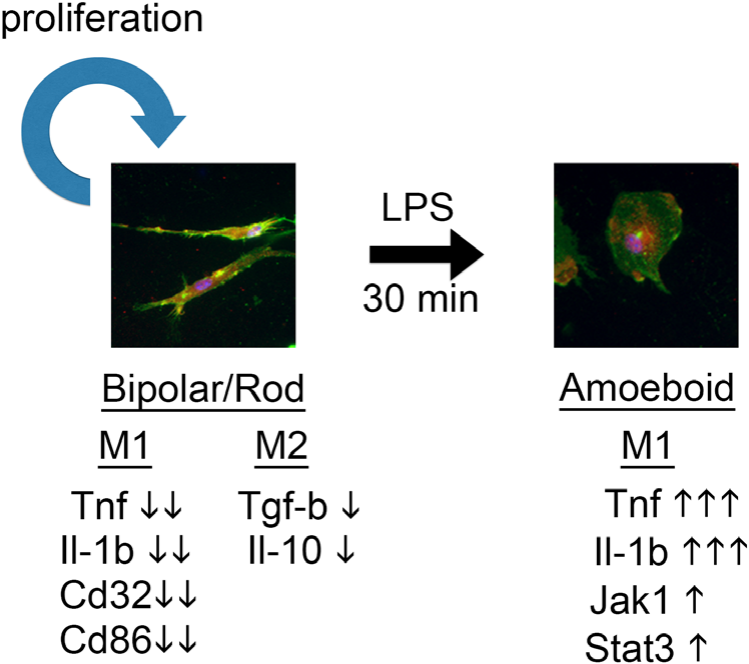
Schematic diagram illustrating the characterization of bipolar/rod-shaped microglia. Bipolar/rod-shaped microglia are highly proliferative and expressed low levels of M1 and M2 markers. Upon LPS treatment, bipolar/rod-shaped microglia rapidly transformed into an amoeboid morphology and became M1-activated.

As summarized in Fig. 6, we demonstrated that scratching PDL/laminin-coated culture dishes enriched the formation of bipolar/rod-shaped microglia, which were highly proliferative and committed to neither an M1 nor an M2 phenotype. Upon LPS treatment, bipolar/rod-shaped microglia rapidly transformed into an amoeboid form and became M1-activated. We propose that our *in vitro* culture system can be used for gene expression studies and further functional characterization of M1/M2 markers in bipolar/rod-shaped microglia, which may exhibit therapeutic potential for neurological diseases.

## Methods

### Animals

The present study was performed in strict accordance with the American Veterinary Medical Association (AVMA) Guidelines on Euthanasia by exposing animals to carbon dioxide. All experimental protocols were approved by the Animal Research Ethics Committee at City University of Hong Kong (Ref. A-0017). We made the best efforts possible to reduce the number of animals used.

### Primary microglia cultures

Primary microglia were isolated (≥99% purity) as previously described^27^. Briefly, cerebral cortices dissected from postnatal day 1 to 3 C57BL/6 mice were trypsinized, mechanically dissociated and seeded on PDL (10 μg/ml)-coated T-75 flasks. The cultures were maintained in DMEM/10% FBS supplemented with macrophage colony stimulating factor (MCSF, 5 ng/ml, Peprotech #315-02). Then, the suspended microglia were retrieved from the culture medium for subsequent experiments.

### Amoeboid-enriched and bipolar/rod-enriched microglia cultures

The 35 mm culture dishes or 8-well chamber slides were coated with PDL (10 μg/ml) followed by laminin (10 μg/ml). The laminin coating maintained the microglia in the amoeboid form^18^; thereafter, we refer to these cultures as “amoeboid-enriched” cultures. For bipolar/rod-enriched microglia cultures, we scratched the PDL/laminin-coated culture dishes or chamber slides using a P200 pipette tip. Then, purified primary microglia were seeded on the scratched culture dishes or chamber slides and were cultured in DMEM/10% FBS at 37°C in a 5% CO_2_ incubator. Scratches generated by different laboratory members produced identical results.

### Cell counting

Cell counting was performed using the ImageJ Cell Counter Plugin. Cells were counted in both the scratched and non-scratched regions and then expressed relative to the area examined (i.e., cells/mm^2^).

### Time-lapse live cell imaging

Microglia were seeded on a scratched PDL/laminin-coated 35 mm culture dish. Time-lapse imaging was performed using an SP5 confocal microscope (Leica) as described previously^53^. Images were captured at 5-minute intervals for more than 14 hours.

### LPS treatment

LPS (1 mg/ml in PBS, Sigma L4516) was added to the culture medium at a final concentration of 10 μg/ml and then applied to the microglia cultures for 30 minutes or 6 hours. Then, total RNA was extracted from the microglia for qPCR analysis.

### Immunostaining

Microglia were fixed using 4% paraformaldehyde at room temperature for 15 minutes. After blocking, the cells were incubated in primary antibodies at 4°C overnight. The primary antibodies used in this study were against IbaI (Wako, #019-19741), CD11b (Serotec, MCA74G) and PCNA (Cell Signaling, #2586). Then, the cells were incubated in the corresponding secondary antibodies conjugated to Alexa Fluor® (Invitrogen) at room temperature for 1 hour. The nuclei were counter-stained with DAPI.

### RNA extraction, reverse transcription and qPCR

RNA was extracted using Trizol reagent (Invitrogen). Reverse transcription was performed using Superscript III First Strand Synthesis SuperMix (Invitrogen). Triplicate qPCR reactions for each sample were performed using the KAPA SYBR Fast qPCR Kit (KAPA) in an ABI 7500 FAST Real-Time PCR system. Ct-values were recorded to calculate the relative fold-change in expression according to the formula 2^-ΔΔCt^; *Gapdh* was used as the standard for normalization. The primer sequences that were used are listed in Table 1.

### Statistical analyses

Statistical analyses were performed using GraphPad Prism 5.0 software. All data are presented as the means ± SEM. Student's *t*-test or one-way ANOVA was used for comparisons; *P* < 0.05 was considered to be significant.

## Author Contributions

W.Y.T. performed the experiments and data analysis. W.Y.T. and C.H.E.M. designed the experiments and wrote the manuscript.

## Supplementary Material

Supplementary Information Supplementary Information

Supplementary Information Supplementary Video S1

Supplementary Information Supplementary Video S2

## Figures and Tables

**Table 1:** qPCR primers used in this study

Gene	Primer sequence (5′ to 3′)
Gapdh	Forward: CATGGCCTTCCGTGTTCCTA
	Reverse: CCTGCTTCACCACCTTCTTGAT
M1 markers	
Tnf	Forward: ACCACGCTCTTCTGTCTACT
	Reverse: GTTTGTGAGTGTGAGGGTCTG
Il-1b	Forward: GAGGACATGAGCACCTTCTTT
	Reverse: GCCTGTAGTGCAGTTGTCTAA
CD32	Forward: AATCCTGCCGTTCCTACTGATC
	Reverse: GTGTCACCGTGTCTTCCTTGAG
CD86	Forward: GACCGTTGTGTGTGTTCTGG
	Reverse: GATGAGCAGCATCACAAGGA
CD16	Forward: TTTGGACACCCAGATGTTTCAG
	Reverse: GTCTTCCTTGAGCACCTGGATC
M2 markers	
Il-10	Forward: GCCTTATCGGAAATGATCCA
	Reverse: TCTCACCCAGGGAATTCAAA
Tgf-β	Forward: TGCGCTTGCAGAGATTAAAA
	Reverse: CGTCAAAAGACAGCCACTCA
Trem2	Forward: CAGTTTCTCCTGCTGCTGAT
	Reverse: CAGTGCTTCAAGGCGTCATA
CD206	Forward: CAAGGAAGGTTGGCATTTGT
	Reverse: CCTTTCAGTCCTTTGCAAGC
Jak1	Forward: CTCTCTGTCACAACCTCTTCGC
	Reverse: TTGGTAAAGTAGAACCTCATGCG
Stat3	Forward: CACCTTGGATTGAGAGTCAAGAC
	Reverse: AGGAATCGGCTATATTGCTGGT

## References

[b1] Nayak D., Roth T. L. & McGavern D. B. Microglia development and function. Annu Rev Immunol 32, 367–402 (2014). 24471431 10.1146/annurev-immunol-032713-120240 PMC5001846

[b2] Zhan Y. *et al.* Deficient neuron-microglia signaling results in impaired functional brain connectivity and social behavior. Nat Neurosci 17, 400–406 (2014). 24487234 10.1038/nn.3641

[b3] Schlegelmilch T., Henke K. & Peri F. Microglia in the developing brain: from immunity to behaviour. Curr Opin Neurobiol 21, 5–10 (2011). 20817438 10.1016/j.conb.2010.08.004

[b4] Saijo K. & Glass C. K. Microglial cell origin and phenotypes in health and disease. Nat Rev Immunol 11, 775–787 (2011). 22025055 10.1038/nri3086

[b5] Ladeby R. *et al.* Microglial cell population dynamics in the injured adult central nervous system. Brain Res Brain Res Rev 48, 196–206 (2005). 15850658 10.1016/j.brainresrev.2004.12.009

[b6] Martinez F. O. & Gordon S. The M1 and M2 paradigm of macrophage activation: time for reassessment. F1000Prime Rep 6, 13 (2014). 24669294 10.12703/P6-13 PMC3944738

[b7] Cherry J. D., Olschowka J. A. & O'Banion M. K. Neuroinflammation and M2 microglia: the good, the bad, and the inflamed. J Neuroinflammation 11, 98 (2014). 24889886 10.1186/1742-2094-11-98 PMC4060849

[b8] Crain J. M., Nikodemova M. & Watters J. J. Microglia express distinct M1 and M2 phenotypic markers in the postnatal and adult central nervous system in male and female mice. J Neurosci Res 91, 1143–1151 (2013). 23686747 10.1002/jnr.23242 PMC3715560

[b9] Durafourt B. A. *et al.* Comparison of polarization properties of human adult microglia and blood-derived macrophages. Glia 60, 717–727 (2012). 22290798 10.1002/glia.22298

[b10] Selenica M. L. *et al.* Diverse activation of microglia by chemokine (C-C motif) ligand 2 overexpression in brain. J Neuroinflammation 10, 86 (2013). 23866683 10.1186/1742-2094-10-86 PMC3726363

[b11] Freilich R. W., Woodbury M. E. & Ikezu T. Integrated expression profiles of mRNA and miRNA in polarized primary murine microglia. PLoS One 8, e79416 (2013). 24244499 10.1371/journal.pone.0079416 PMC3823621

[b12] Prinz M. & Priller J. Microglia and brain macrophages in the molecular age: from origin to neuropsychiatric disease. Nat Rev Neurosci 15, 300–312 (2014). 24713688 10.1038/nrn3722

[b13] Stence N., Waite M. & Dailey M. E. Dynamics of microglial activation: a confocal time-lapse analysis in hippocampal slices. Glia 33, 256–266 (2001). 11241743

[b14] Suzumura A., Marunouchi T. & Yamamoto H. Morphological transformation of microglia in vitro. Brain Res 545, 301–306 (1991). 1907214 10.1016/0006-8993(91)91302-h

[b15] Szabo M. & Gulya K. Development of the microglial phenotype in culture. Neuroscience 241, 280–295 (2013). 23535251 10.1016/j.neuroscience.2013.03.033

[b16] Hanisch U. K. & Kettenmann H. Microglia: active sensor and versatile effector cells in the normal and pathologic brain. Nat Neurosci 10, 1387–1394 (2007). 17965659 10.1038/nn1997

[b17] Nimmerjahn A., Kirchhoff F. & Helmchen F. Resting microglial cells are highly dynamic surveillants of brain parenchyma in vivo. Science 308, 1314–1318 (2005). 15831717 10.1126/science.1110647

[b18] Chamak B. & Mallat M. Fibronectin and laminin regulate the in vitro differentiation of microglial cells. Neuroscience 45, 513–527 (1991). 1663599 10.1016/0306-4522(91)90267-r

[b19] Taylor S. E., Morganti-Kossmann C., Lifshitz J. & Ziebell J. M. Rod microglia: a morphological definition. PLoS One 9, e97096 (2014). 24830807 10.1371/journal.pone.0097096 PMC4022629

[b20] Ziebell J. M., Taylor S. E., Cao T., Harrison J. L. & Lifshitz J. Rod microglia: elongation, alignment, and coupling to form trains across the somatosensory cortex after experimental diffuse brain injury. J Neuroinflammation 9, 247 (2012). 23111107 10.1186/1742-2094-9-247 PMC3526458

[b21] Zhan X., Kim C. & Sharp F. R. Very brief focal ischemia simulating transient ischemic attacks (TIAs) can injure brain and induce Hsp70 protein. Brain Res 1234, 183–197 (2008). 18708034 10.1016/j.brainres.2008.07.094 PMC2670998

[b22] Boche D., Perry V. H. & Nicoll J. A. Review: activation patterns of microglia and their identification in the human brain. Neuropathol Appl Neurobiol 39, 3–18 (2013). 23252647 10.1111/nan.12011

[b23] Graeber M. B. Changing face of microglia. Science 330, 783–788 (2010). 21051630 10.1126/science.1190929

[b24] Wierzba-Bobrowicz T. *et al.* Morphological analysis of active microglia--rod and ramified microglia in human brains affected by some neurological diseases (SSPE, Alzheimer's disease and Wilson's disease). Folia Neuropathol 40, 125–131 (2002). 12572918

[b25] Imai Y. & Kohsaka S. Intracellular signaling in M-CSF-induced microglia activation: role of Iba1. Glia 40, 164–174 (2002). 12379904 10.1002/glia.10149

[b26] Roy A., Fung Y. K., Liu X. & Pahan K. Up-regulation of microglial CD11b expression by nitric oxide. J Biol Chem 281, 14971–14980 (2006). 16551637 10.1074/jbc.M600236200 PMC1963414

[b27] Griffin R. S. *et al.* Complement induction in spinal cord microglia results in anaphylatoxin C5a-mediated pain hypersensitivity. J Neurosci 27, 8699–8708 (2007). 17687047 10.1523/JNEUROSCI.2018-07.2007 PMC6672952

[b28] Giulian D. Ameboid microglia as effectors of inflammation in the central nervous system. J Neurosci Res 18, 155–171, 132–153 (1987). 10.1002/jnr.490180123 3500323

[b29] Giulian D. & Baker T. J. Characterization of ameboid microglia isolated from developing mammalian brain. J Neurosci 6, 2163–2178 (1986). 3018187 10.1523/JNEUROSCI.06-08-02163.1986 PMC6568755

[b30] Giulian D., Baker T. J., Shih L. C. & Lachman L. B. Interleukin 1 of the central nervous system is produced by ameboid microglia. J Exp Med 164, 594–604 (1986). 3487617 10.1084/jem.164.2.594 PMC2188228

[b31] Lenz K. M., Nugent B. M., Haliyur R. & McCarthy M. M. Microglia are essential to masculinization of brain and behavior. J Neurosci 33, 2761–2772 (2013). 23407936 10.1523/JNEUROSCI.1268-12.2013 PMC3727162

[b32] Fischer H. G., Bielinsky A. K., Nitzgen B., Daubener W. & Hadding U. Functional dichotomy of mouse microglia developed in vitro: differential effects of macrophage and granulocyte/macrophage colony-stimulating factor on cytokine secretion and antitoxoplasmic activity. J Neuroimmunol 45, 193–201 (1993). 8331161 10.1016/0165-5728(93)90180-7

[b33] Nakamura Y., Si Q. S. & Kataoka K. Lipopolysaccharide-induced microglial activation in culture: temporal profiles of morphological change and release of cytokines and nitric oxide. Neurosci Res 35, 95–100 (1999). 10616913 10.1016/s0168-0102(99)00071-1

[b34] Welser-Alves J. V. & Milner R. Microglia are the major source of TNF-alpha and TGF-beta1 in postnatal glial cultures; regulation by cytokines, lipopolysaccharide, and vitronectin. Neurochem Int 63, 47–53 (2013). 23619393 10.1016/j.neuint.2013.04.007 PMC3819935

[b35] Yates S. L. *et al.* Amyloid beta and amylin fibrils induce increases in proinflammatory cytokine and chemokine production by THP-1 cells and murine microglia. J Neurochem 74, 1017–1025 (2000). 10693932 10.1046/j.1471-4159.2000.0741017.x

[b36] Pannell M., Szulzewsky F., Matyash V., Wolf S. A. & Kettenmann H. The subpopulation of microglia sensitive to neurotransmitters/neurohormones is modulated by stimulation with LPS, interferon-gamma, and IL-4. Glia 62, 667–679 (2014). 24504982 10.1002/glia.22633

[b37] Qin H. *et al.* Molecular mechanism of lipopolysaccharide-induced SOCS-3 gene expression in macrophages and microglia. J Immunol 179, 5966–5976 (2007). 17947670 10.4049/jimmunol.179.9.5966

[b38] Jonas R. A., Yuan T. F., Liang Y. X., Jonas J. B., Tay D. K. & Ellis-Behnke R. G. The spider effect: morphological and orienting classification of microglia in response to stimuli in vivo. PLoS One 7, e30763 (2012). 22363486 10.1371/journal.pone.0030763 PMC3283598

[b39] Huang C. *et al.* JAK2-STAT3 signaling pathway mediates thrombin-induced proinflammatory actions of microglia in vitro. J Neuroimmunol 204, 118–125 (2008). 18710787 10.1016/j.jneuroim.2008.07.004

[b40] Liva S. M., Kahn M. A., Dopp J. M. & de Vellis J. Signal transduction pathways induced by GM-CSF in microglia: significance in the control of proliferation. Glia 26, 344–352 (1999). 10383053 10.1002/(sici)1098-1136(199906)26:4<344::aid-glia8>3.0.co;2-l

[b41] Przanowski P. *et al.* The signal transducers Stat1 and Stat3 and their novel target Jmjd3 drive the expression of inflammatory genes in microglia. J Mol Med (Berl) 92, 239–254 (2014). 24097101 10.1007/s00109-013-1090-5 PMC3940857

[b42] Yang X. *et al.* The role of the JAK2-STAT3 pathway in pro-inflammatory responses of EMF-stimulated N9 microglial cells. J Neuroinflammation 7, 54 (2010). 20828402 10.1186/1742-2094-7-54 PMC2945324

[b43] Spielmeyer W. Histopathologie Des Nervensystems. (Verlag von Julius Springer, Berlin, 1922).

[b44] Denes A. *et al.* Proliferating resident microglia after focal cerebral ischaemia in mice. J Cereb Blood Flow Metab 27, 1941–1953 (2007). 17440490 10.1038/sj.jcbfm.9600495

[b45] Lalancette-Hebert M., Gowing G., Simard A., Weng Y. C. & Kriz J. Selective ablation of proliferating microglial cells exacerbates ischemic injury in the brain. J Neurosci 27, 2596–2605 (2007). 17344397 10.1523/JNEUROSCI.5360-06.2007 PMC6672496

[b46] Amat J. A., Ishiguro H., Nakamura K. & Norton W. T. Phenotypic diversity and kinetics of proliferating microglia and astrocytes following cortical stab wounds. Glia 16, 368–382 (1996). 8721677 10.1002/(SICI)1098-1136(199604)16:4<368::AID-GLIA9>3.0.CO;2-W

[b47] Glass C. K., Saijo K., Winner B., Marchetto M. C. & Gage F. H. Mechanisms underlying inflammation in neurodegeneration. Cell 140, 918–934 (2010). 20303880 10.1016/j.cell.2010.02.016 PMC2873093

[b48] Krause D. L. & Muller N. Neuroinflammation, microglia and implications for anti-inflammatory treatment in Alzheimer's disease. Int J Alzheimers Dis 2010 (2010). 10.4061/2010/732806 PMC2925207 20798769

[b49] Smith J. A., Das A., Ray S. K. & Banik N. L. Role of pro-inflammatory cytokines released from microglia in neurodegenerative diseases. Brain Res Bull 87, 10–20 (2012). 22024597 10.1016/j.brainresbull.2011.10.004 PMC9827422

[b50] Sawada M., Suzumura A., Hosoya H., Marunouchi T. & Nagatsu T. Interleukin-10 inhibits both production of cytokines and expression of cytokine receptors in microglia. J Neurochem 72, 1466–1471 (1999). 10098850 10.1046/j.1471-4159.1999.721466.x

[b51] Cao T., Thomas T. C., Ziebell J. M., Pauly J. R. & Lifshitz J. Morphological and genetic activation of microglia after diffuse traumatic brain injury in the rat. Neuroscience 225, 65–75 (2012). 22960311 10.1016/j.neuroscience.2012.08.058 PMC3489473

[b52] Minghetti L. Role of inflammation in neurodegenerative diseases. Curr Opin Neurol 18, 315–321 (2005). 15891419 10.1097/01.wco.0000169752.54191.97

[b53] Ho Y. M. *et al.* A lysosome-specific two-photon phosphorescent binuclear cyclometalated platinum(II) probe for in vivo imaging of live neurons. Chem Commun (Camb) 50, 4161–4163 (2014). 24418895 10.1039/c3cc48934e

