# Supplementary material for: Bipolar/rod-shaped microglia are proliferating microglia with distinct M1/M2 phenotypes
Source: Sci Rep. 2014 Dec 2;4:7279. doi: 10.1038/srep07279 (PMC4250916; doi:10.1038/srep07279)
Supplement: Supplementary Information [file srep07279-s1.pdf]

Supplementary Information

Bipolar/rod-shaped microglia are proliferating microglia with distinct M1/M2 phenotypes

Wing Yip Tam[1], Chi Him Eddie Ma*[1, 2, 3]

[1] Department of Biomedical Sciences, [2] Centre for Biosystems, Neuroscience, and Nanotechnology, [3] State Key Laboratory in Marine Pollution, City University of Hong Kong, Tat Chee Avenue, Hong Kong

*Corresponding author: eddiema@cityu.edu.hk

This file includes:

Supplementary Figures S1

Other Supplementary Materials for this manuscript includes the following:

Supplementary Video S1 and S2 | Migration of microglia toward the scratched area. Time-lapse live cell imaging showed the migration of microglia toward the scratched area during the first 14 hours after cell seeding.

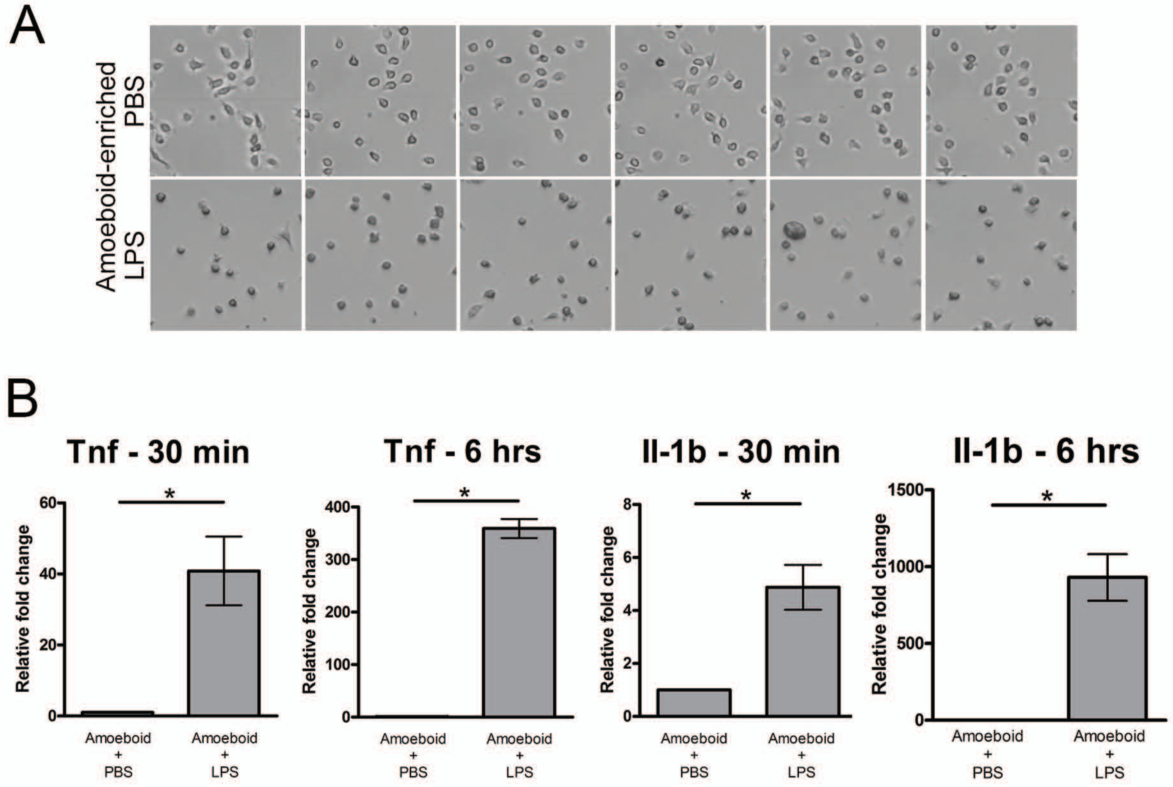

Supplementary Figure S1 | Amoeboid microglia responded to M1 activation using LPS. (a) Amoeboid microglia maintained their amoeboid morphology in response to LPS or PBS treatment. **(b)** qPCR showed significant upregulation of Tnf and Il-1b after 30 minutes and 6 hours of LPS stimulation compared with PBS treatment (n=3 or 4). *P < 0.05 based on Student's t-test.
